# Meta-analysis of health state utility values measured by EuroQol 5-dimensions (EQ5D) questionnaire in Chinese women with breast cancer

**DOI:** 10.1186/s12885-021-09140-5

**Published:** 2022-01-10

**Authors:** Tamlyn Rautenberg, Brent Hodgkinson, Ute Zerwes, Martin Downes

**Affiliations:** 1 Centre for Applied Health Economics, Griffith University, Brisbane, Australia; 2 Assessment in Medicine GmbH, Berlin, Germany

**Keywords:** evidence synthesis, meta-analysis, health state utility value, EQ-5D, breast cancer, China

## Abstract

**Background:**

To synthesise EQ5D health state utility values in Chinese women with breast cancer for parameterising a cost utility model.

**Methods:**

Eligible studies had to report health state utility values measured by EQ-5D in Chinese women diagnosed with breast cancer. Risk of bias was assessed using the Newcastle Ottawa Scale (NOS). Data from single arm studies was pooled using meta-analysis of single proportions to provide overall point estimates and 95% confidence intervals for fixed and random effects models using the inverse variance and Der Simonian-Laird methods respectively. Heterogeneity was evaluated using the I^2^ statistic and sensitivity analysis and meta-regression were conducted.

**Results:**

Five papers were included, when all studies were combined (n = 4,100) the mean utility (95% confidence interval) for random effects model was 0.83 (0.78, 0.89); for TNM 0-1 0.85 (0.75, 0.95); for TNM II 0.85 (0.78, 0.93); for TNM III 0.83 (0.77, 0.90) and for TNM IV 0.73 (0.63, 0.82).The utility of patients in State P (first year after primary breast cancer) 0.84 (0.80, 0.88); in State R (first year after recurrence) 0.73 (0.69, 0.76), in State S (second and following years after primary breast cancer or recurrence) 0.88 (0.83, 0.92); and in State M (metastatic disease) 0.78 (0.74, 0.82). Mean utility for duration since diagnosis 13 to 36 months was 0.88 (0.80, 0.96, I^2^ =95%); for 37 to 60 months 0.89 (0.82, 0.96, I^2^ =90%); for more than 60 months 0.86 (0.76, 0.96, I^2^ =90%). Mean utility for chemotherapy was 0.86 (0.79, 0.92, I^2^ =97%); for radiotherapy 0.83 (0.69, 0.96, I^2^ =97%); surgery 0.80 (0.69, 0.91, I^2^ =98%); concurrent chemo-radiation 0.70 (0.60, 0.81, I^2^ =86%) and endocrine therapy 0.90 (0.83, 0.97, I^2^ =91%). Conclusion: This study synthesises the evidence for health state utility values for Chinese women with breast cancer which is useful to inform cost utility models.

**Supplementary Information:**

The online version contains supplementary material available at 10.1186/s12885-021-09140-5.

## Background

Breast cancer has now overtaken lung cancer to become the most commonly diagnosed cancer in women in China [1, 2]. The number of breast cancer cases in China increased from 304,000 in 2015 to 413,000 in 2020 [2, 3]. Breast cancer impacts on physical and emotional wellbeing which, taken together, provide an indication of patient’s quality of life. Quality of life is an important indicator of the impact of health status on patients’ wellbeing. It is usually an important component of measuring patients’ health outcomes and can be used to inform decision making.

This is particularly relevant now, as pharmacoeconomics is playing an increasingly important role in China, evidenced by the recent 2020 guideline that recommends cost-utility analysis and quality adjusted life years for pharmacoeconomic analysis [4, 5]. It also advocates indirect measures like the five-level EuroQoL Group's five-dimension questionnaire (EQ-5D) using the scoring algorithm for Chinese population, over direct measures [4]. The EQ-5D is a widely used and internationally validated instrument measuring utility on a scale between zero (death) and one (full health). Respondents value their own health against five domains (mobility, self-care, usual activity, pain/discomfort and anxiety/depression) and grade their response according to three (3L) or, more recently, five (5L) levels of each domain. Values are converted to utilities using a reference population’s value set. A valuation set is available for China and studies confirm that the Chinese version provides comparable results to the English version [6, 7]. Importantly, the EQ-5D has demonstrated validity, responsiveness, and reliability in assessing health outcomes specifically for breast cancer patients [8, 9].

Cost utility analyses require health state utility values. There are few studies that measure health state utility values of Chinese women with breast cancer and these few studies, when taken together may provide an estimate of effect which can be useful to inform decisions making. To parameterise a cost utility model when multiple primary studies are available, performing an evidence synthesis with a single arm pooling (or meta-analysis), can provide an acceptable estimate and is in keeping with best practice recommendations for modelling [10]. In the context of the burden of breast cancer in China, the increasing role of Pharmacoeconomics and the need to parameterise a cost-utility model, the following meta-analysis was undertaken.

## Methods

Literature search strategy: Pubmed (Medline, Pubmed central and Bookshelf) was searched (28.05.2020) from January 2002 to May 2020. The Cochrane and Centre for Reviews and Dissemination databases were searched (Cochrane Database of Systematic Reviews, Database of Abstracts of Reviews of Effectiveness (DARE), Cochrane Central Register of Clinical Trials, Health Technology Assessment (HTA), Economic Evaluation (NHS EED) was searched on (20.11.2020). The literature search strategy was replicated on 20.08.2021 for the preceding year, no new results were identified. Citation snowballing was used to identify additional papers. The search terms in Table 1 were combined using Boolean operators.

**Table 1:** Search terms used (all databases)

Element of clinical question	Search terms
Population Mesh terms Humans, Women, Quality of life, Quality Adjusted Life Years, Breast neoplasms	Breast neoplasm*(TI/AB), Breast cancer* (TI/AB), Breast carcinoma* (TI/AB), Breast tumour* (TI/AB), Mammary cancer* (TI/AB), Mammary neoplasm* (TI/AB),Mammary carcinoma* (TI/AB), Mammary tumor* (TI/AB) China (TI/AB), Chinese (TI/AB)
Intervention/ comparator/outcome(s)	EuroQoL, EuroQoL-5D, EQ-5D, Health utilities [tw], Health-state utilities [tw] Health Utilities Index [tw], HUI2, HUI3, Utility score* [tw], Utility value*[tw], Utilities [tw] NOT (clinical utilities [tw] OR Diagnostic utilities [tw]), Utility [tw] NOT (clinical utility [tw] OR diagnostic utility [tw]), Standard gamble [tw], Time trade-off [tw], TTO [tiab]

*indicates a truncated term; AB = abstract; TI = title; tw = text word

Eligibility criteria: pre-specified selection criteria were applied. Included studies had to be conducted in China (or Asian countries with >80% Chinese participants), had to use the EQ5D instrument to measure Quality of Life and had to report health state utility values of women with breast cancer. Included studies needed to report essential summary measures required for meta-analysis (mean utility and variance).

Study selection: Titles and abstracts were screened by two trained, independent reviewers (TR, BH). Titles/abstracts of unclear eligibility were included at this stage. Full texts were retrieved and reviewed by two independent reviewers (TR, BH). Discrepancies were resolved via an independent third reviewer (MD).

Data extraction and quality assessment: Data was extracted and verified independently by two reviewers (UZ, BH). Data fields extracted included study location and design, population demographics, the description of the related health state, sample size, mean utility and variance. Risk of bias was assessed using the Newcastle Ottawa Scale (NOS) which is applicable for assessing the level of bias in single-arm non-randomized studies [11]. The NOS scale is comprised of five items addressing subject selection and attrition, reporting all intended outcomes, and any other relevant considerations, rating each item as either at low, unclear or high risk of bias [11]. NOS has been applied to other single arm studies including a systematic review by the Agency for Healthcare Research and Quality and a review of chloroquine/hydroxychloroquine effectiveness for COVID-19 [12, 13]. The literature review was conducted and reported in compliance with Cochrane and PRISMA guidelines [14, 15].

Meta-analysis: The meta-analysis was considered feasible if two or more studies reported utility for breast cancer and was conducted according to international recommendations [16]. Data from the single arm studies were pooled and meta-analysis of single proportions (metaprop) was conducted from the R meta package (version 3.6.1) to provide point estimates and 95% confidence intervals (CI) for fixed and random effects models (using the inverse variance method and Der Simonian-Laird method respectively) [17]. Heterogeneity was evaluated using the I^2^ statistic and where possible meta-regression of covariates was conducted in OpenMeta [18]. Meta-analysis was performed for all patients, according to Tumor Nodal Metastases (TNM) staging system and PRSM states. State P was defined as first year after diagnosis of primary breast cancer; State R as first year after recurrence; State S as second and following years after primary breast cancer/recurrence; State M as metastatic breast cancer.

## Results

**Fig. 1:**
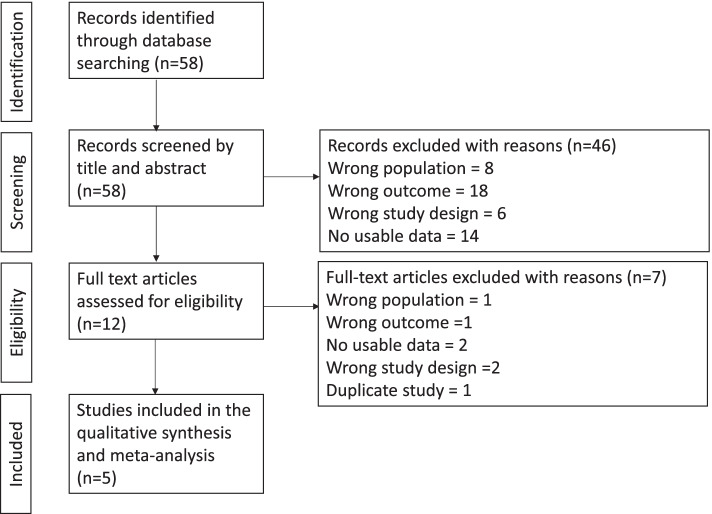
PRISMA diagram describing the results of the literature search and the reasons for study exclusion

Study selection: A total of 58 papers were identified and 53 of which were excluded during title/abstract screening and full text assessment. In total, nine papers were excluded because they focused on incorrect population (conducted outside China/did not include more than 80% of Chinese respondents/focus on other cancers). Nineteen studies did not report quality of life data or derived it according to other disease-specific or generic instruments. Sixteen studies did not report sufficient measures of effect and variance and had no usable data. Eight studies were the incorrect study design, six of which were economic evaluations/cost effectiveness studies and two of which were pilot studies. One duplicate study was identified which reports the same patient sample (personal communication) and values, the most recent paper has been included in the analysis [19, 20]. Five studies met the eligibility criteria [20–24]. A PRISMA flow diagram is shown in Fig 1.

Study overview: Four of the five studies were full published papers [20, 21, 23, 24] and one was in poster format and provided all data required [22]. Yang (2020) performed a survey of 446 patients in a tertiary oncology hospital in China between November 2017 and May 2018 [20]. Li et al (2019) measured utility in a sample of 608 breast cancer patients who underwent inpatient treatment at a single hospital in China between October 2014 to February 2015 [23]. Ou (2019) measured utility in a cross-sectional survey of 193 Chinese patients in Taiwan in 2017 [24]. Wang (2018) reported on utility scores from a survey of 2,626 breast cancer patients across 12 provinces in China from September 2013 to December 2014 [22]. The oldest study by Cheung (2014) was a cross-sectional survey in two cancer centres in Singapore of a sample of 238 Asian inpatients and outpatients with histologically confirmed breast cancer in 2014 [21]. Wang used the three-level version of the EQ5D, all other studies used the five-level version. Cheung (2014) used a Japanese value set as the study was conducted prior to the publication of a Chinese data set [21]. A summary of key differences is shown in Table 2.

**Table 2:** Key differences in studies

Study	Region	Year of the data	Sample size	Levels of EQ-5D	Value set
Li [23]	China	October 2014 to February 2015	608	Five	Chinese 5L value set Luo 2017 – Time trade off (TTO)
Ou [24]	**Taiwan (100% Chinese)**	2017 (specific time period not stated)	193	Five	Chinese 5L value set Luo 2017 – Time trade off (TTO)
Wang [22]	China	September 2013 to December 2014	2626	**Three**	Chinese 3L value set Liu 2014 – – Time trade off (TTO)
Cheung [21]	**Singapore (81% Chinese)**	Prior to 2013 (specific time period not stated [7])	238	Five	**Japanese value set** (only available Asian value set for the cross-walk project at that time).
Yang [20]	China	November 2017 to May 2018.	446	Five	Chinese 5L value set Luo 2017 – Time trade off (TTO)

Quality assessment: With respect to sampling bias: Ou is considered to be at high risk of being non-representative of the general population because it includes patients referred to a specialist clinic with hormone receptor positive and human epidermal growth factor receptor 2 negative subtypes of breast cancer only. Furthermore, although the study includes 100% Chinese patients, it is conducted in Taiwan. Cheung is considered to be at moderate risk of bias because it includes 81% of Chinese patients from Singapore [21, 24]..

Four of the studies were judged to be at low risk of attrition bias: Wang did not report the number of participants that utility values were elicited from and Ou [22] neglected to account for dropouts (5.7% of initial study population )[24]. In the Cheung study, 280 patients consented to the study, 39 did not self-administer the questionnaire and 3 patients were excluded due to missing values in two measuring instruments beyond imputation by the half rule [21]. In the Li study, 621 patients were interviewed, 11 did not complete the questionnaire, 1 did not answer a TTO question, 1 did not complete the clinical chart leaving a sample of 608 participants and 1 other participant with missing values [23]. Yang was considered to be at moderate risk of bias because it did not report information on attrition.

Four of the studies were considered at low risk of bias with respect to timing of administering the EQ5D. Li and Yang had similar distribution of patients according to time since diagnosis. Approximately a third were less than 12 months since diagnosis (31% vs. 31%); a third were 13-36 months since diagnosis (32% vs 33%); remainder of patients were distributed evenly across 37 to 60 months (19% vs 17%) and more than 61 months since diagnosis (18% vs 20%) for Li and Yang respectively. Ou study was considered to be at moderate risk of bias because more patients were longer since diagnosis compared to Li and Yang: 34% of patients were within 36 months of diagnosis, 39% were 37-60 months, 14% were 84-108 months and 13% were more than 108 months since diagnosis. According to Wang, 9% of respondents were pre-treatment, 64% were having treatment, 21% were after treatment and 6 % were during follow-up. Cheung did not explicitly state duration, however 48% were undergoing treatment, 33% were having palliative treatment and 19% were having follow-up or no treatment. Both studies were considered to be low risk of bias. No issues of selective reporting were raised, except for Yang due to the failure to report details on attrition. The risk of bias assessed by NOS is shown in Table 3.

**Table 3:** Newcastle Ottawa Scale Risk of Bias Ratings

Newcastle Ottawa Scale Question	First author, year				
	Cheung 2014 [21]	Wang 2018 [22]	Li 2019 [23]	Ou 2019 [24]	Yang 2020 [20]
Sampling: Were the subjects in the study representative of the entire population from which they were recruited?	Moderate	Low	Low	High	Low
Measurement: Incomplete outcome data: attrition bias due to amount, nature or handling of incomplete outcome data	Low	Low	Low	Low	Moderate
Measurement: Do the analyses adjust for different lengths of follow-up of patients?	Low	Low	Low	Moderate	Low
Measurement: Selective reporting: reporting bias due to selective outcome reporting	Low	Low	Low	Low	Moderate
Additional bias: Bias due to problems not covered elsewhere.	Low	Low	Low	Low	Low

The Newcastle-Ottawa Scale (NOS) [11].

Study characteristics: The average age of study participants ranged between 48 and 55 years, with the majority of participants being married (range 71% to 92%). Participants from Taiwan [24] and Singapore [21] were more highly educated (42% and 33% respectively) than participants recruited in China (14%-25%) [20, 23].

Health state utility values: Utility values from the five included studies are shown in Table 4.

**Table 4:** Summary of EQ5D utility scores included in studies

Outcome	Yang 2020 [20]	Li 2019 [23]	Ou 2019 [24]	Wang 2018 [22]	Cheung 2014 [21]
	n = 446	n = 608	n = 193	n = 2626	n = 238
EQ-5D-5L	Mean ± SD	Mean ± SD	Mean ± SD	Mean (95%CI)	Mean ± SD
Mean	0.86 ± 0.19	0.83 ± 0.18	0.92 ± 0.09 (n=182)	0.78 (0.77, 0.79)	0.77 ± 0.163
Stage or TNM stage					
Stage/TNM 0	nr	nr	0.93 ± 0.06 (n=22)	nr	nr
Stage/TNM I	nr	nr	0.94 ± 0.06 (n=48)	0.79 (0.77, 0.81) (n=498)	nr
Stage/TNM 0&I	nr	0.83 ± 0.17 (n=175)	nr	nr	nr
Stage/TNM II	nr	0.86 ± 0.18 (n=142)	0.91 ± 0.10 (n=65)	0.79 (0.78, 0.80) (n=1,234)	nr
Stage/TNM III	nr	0.82 ± 0.18 (n=218)	0.91 ± 0.12 (n=37)	0.77 (0.76, 0.79) (n=556)	nr
Stage/TNM IV	nr	0.78 ± 0.22 (n=73)	0.91 ± 0.03 (n=3)	0.69 (0.65, 0.72) (n=224)	nr
State					
State P	0.81 ± 0.23 (n=125)	0.86 ± 0.17 (n=157)	nr	nr	nr
State S	0.90 ± 0.12 (n=258)	0.85 ± 0.16 (n=245)	nr	nr	nr
State R	0.78 ± 0.31 (n=20)	0.72 ± 0.16 (n=65)	nr	nr	nr
State M	0.74 ± 0.27 (n=43)	0.79 ± 0.23 (n=141)	nr	nr	nr
Time since diagnosis					
≤12 months	0.81 (nr) (n=133)	0.81 ±0.18 (n=190)	nr	nr	nr
13-36 months	0.89 (nr) (n=147)	0.84 ±0.18 (n=197)	0.92 ±0.08 (n=66)^a^	nr	nr
37-60 months	0.91 (nr) (n=78)	0.85 ± 0.20 (n=112)	0.92 ± 0.09 (n=74)b	nr	nr
≥ 61 months	0.83 (nr) (n=88)	0.81 ± 0.17 (n=109)	0.94 ± 0.10 (n=27)c 0.89 ± 0.10 (n=25)d	nr	nr
Treatment regimen					
Chemotherapy	nr	0.86 ±0.17 (n=288)	0.91 ±0.10 (n=135)	0.80 (0.78,0.81) (n=689)	nr
Surgery & chemotherapy	nr	0.71 ±0.15 (n=69)	nr	0.79 (0.78,0.80) (n=849)e 0.78 (0.75,0.81) (n=179)f	nr
Concurrent chemoradiotherapy	nr	0.65 ±0.14 (n=26)	nr	0.76 (0.70,0.82) (n=47)	nr
Radiotherapy	nr	0.79 ±0.11 (n=14)	0.94 ±0.08 (n=71)g 0.91 ±0.10 (n=108)h	nr	nr
Endocrine therapy	0.88 (nr) (n=307)	0.86 ±0.16 (n=73)	0.93 ±0.08 (n=107)i 0.91 ±0.10 (n=79)j	nr	nr

TNM = Tumor, Nodes, Metastases. nr=not reported. a reported as <36 months; b reported as 3-6 years; c reported as 7-9 years; d reported as >9 years; e surgery and postoperative chemotherapy, f surgery & neoadjuvant chemotherapy; g partial mastectomy; h total mastectomy; i Tamoxifen, j Aromatose inhibitors.

Meta-analysis: When mean utility from all patients in the five studies are combined (n = 4,100) the mean utility (95% confidence interval) is 0.83 (0.78, 0.89) and 0.82 (0.81, 0.83, I^2^ = 99%) for random and fixed effects models respectively shown in Table 5.

**Table 5:**
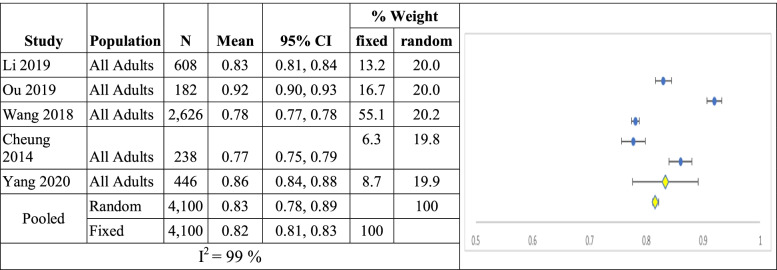
Summary of single arm meta-analyses of studies of all breast cancer patients

CI = confidence interval

When studies were combined according to TNM stage, for TNM 0-1 and 1 (n=721) mean utility was 0.85 (0.75, 0.95) and 0.85 (0.84, 0.86, I^2^ =98%); for TNM II (n=1,441) 0.85 (0.78, 0.93) and 0.81 (0.80,0.82, I^2^ =97%); for TNM III (n=811) 0.83 (0.77, 0.90) and 0.80 (0.79,0.81, I^2^ =95%) and TNM IV (n=297) 0.73 (0.63, 0.82) and 0.71 (0.68, 0.73, I^2^ =89%) for random and fixed effects models respectively shown in Table 6.

**Table 6:**
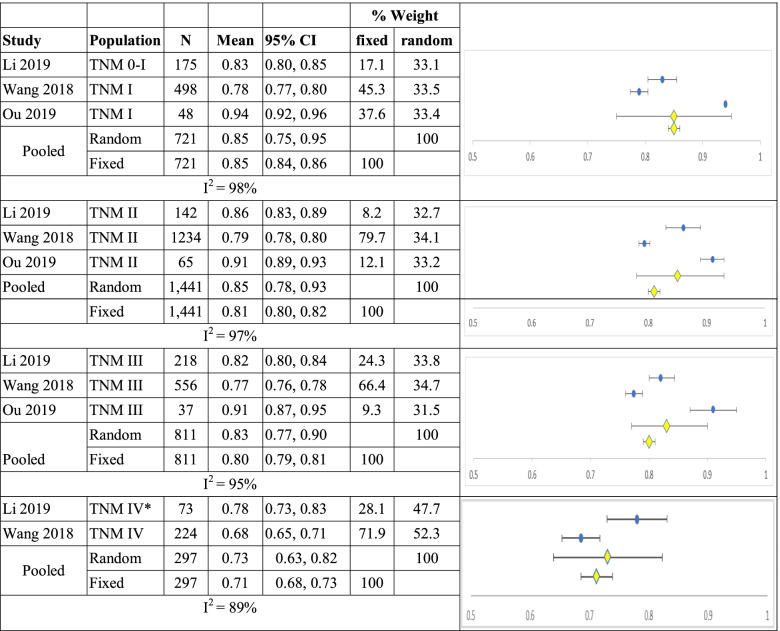
Summary of single arm meta-analyses of studies of breast cancer patients by TNM classification.

*Ou study included only 3 respondents therefore was excluded from this analysis, CI = confidence interval; TNM = classification of malignant tumours (T = size of tumor, N = spread to lymph nodes, M = metastasis)

Li and Yang reported data by cancer state, mean utility for state P (n=282) was 0.84 (0.80, 0.88) and 0.85 (0.82, 0.87, I^2^ =71%); for state S (n=503) 0.88 (0.83, 0.92) and 0.88 (0.87,0.89, I^2^ =97%); for state R (n=85) 0.73 (0.69, 0.76) and 0.73 (0.69, 0.76, I^2^ =0%); for state M (n=184) 0.78 (0.74, 0.82) and 0.78 (0.75, 0.82, I^2^ =17%) for random and fixed effects models respectively shown in Table 7.

**Table 7:**
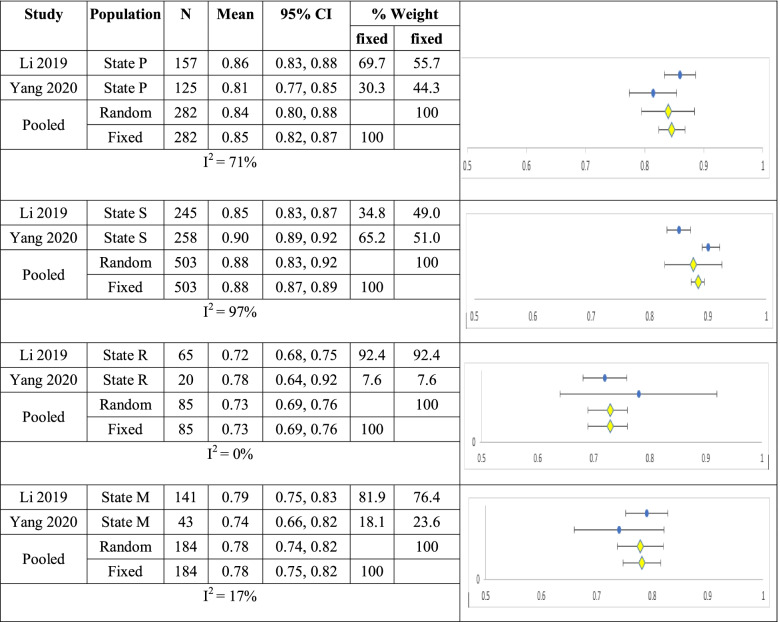
Summary of single arm meta-analyses of studies of breast cancer patients by PSRM State

CI – confidence interval; State P, first year after primary breast cancer; State R, first year after recurrence; State S, second and following years after primary breast cancer/recurrence; State M, metastatic breast cancer.

Li and Yang reported data according to time since diagnosis. Mean utility for duration 13 to 36 months (n=263) was 0.88 (0.80, 0.96) and 0.89 (0.88, 0.91, I^2^ =95%); for 37 to 60 months (n=186) 0.89 (0.82, 0.96) and 0.90 (0.89,0.92, I^2^ =90%); for more than 60 months (n=161) 0.86 (0.76, 0.96) and 0.84 (0.81, 0.85, I^2^ =90%) for random and fixed effects models respectively shown in Table 8.

**Table 8:**
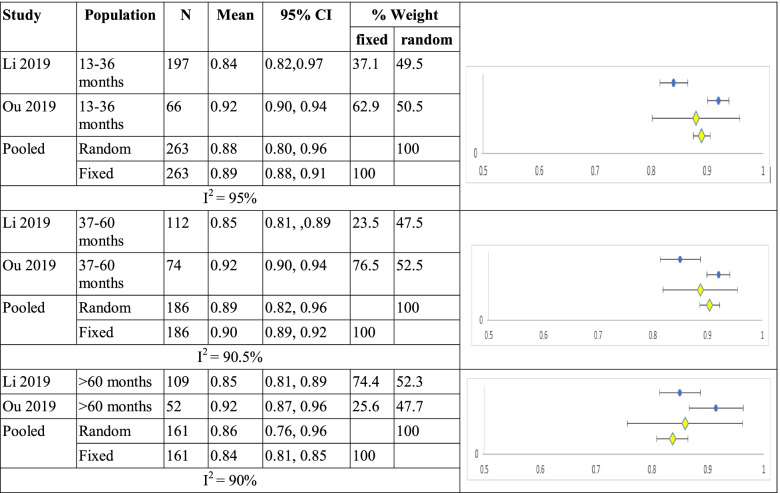
Summary of single arm meta-analyses of studies of breast cancer patients by duration since diagnosis

Li, Ou and Wang reported data according to treatment regimen. Mean utility for chemotherapy (n=850) was 0.86 (0.79, 0.92) and 0.85 (0.84, 0.86, I^2^ =97%); for radiotherapy (n=332) 0.83 (0.69, 0.96) and 0.90 (0.88,0.91, I^2^ =97%) and for surgery (n=891) 0.80 (0.69, 0.91) and 0.77 (0.76, 0.79, I^2^ =98%). Li and Wang reported concurrent chemo-radiation therapy (n=73) 0.70 (0.60, 0.81) and 0.70 (0.66, 0.74, I^2^ =86%). Li and Ou reported results for endocrine therapy (n=180) 0.90 (0.83, 0.97) and 0.92 (0.91, 0.93, I^2^ =91%) for random and fixed effects models respectively shown in Table 9.

**Table 9:**
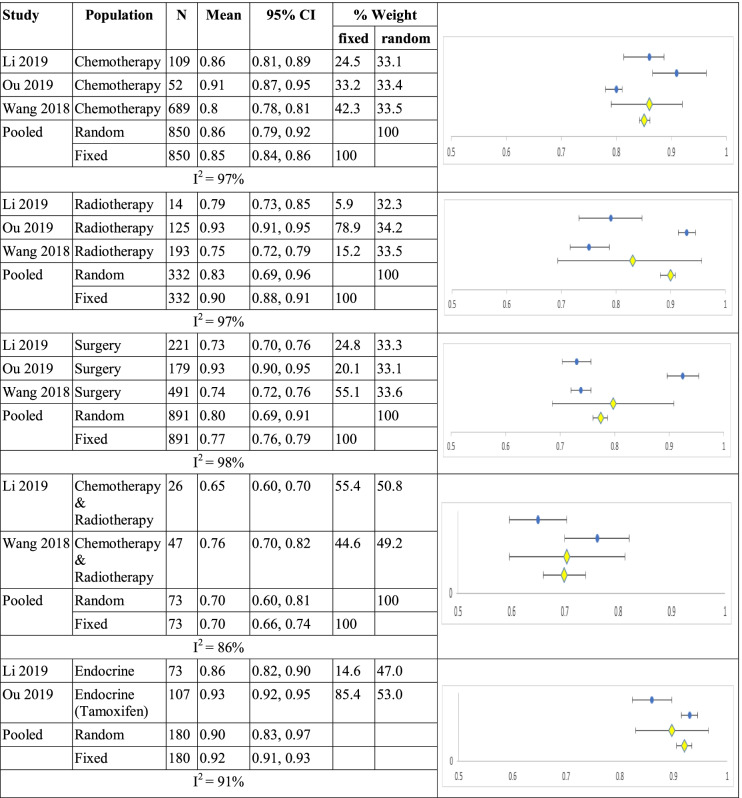
Summary of single arm meta-analyses of studies of breast cancer patients by treatment regimen

Meta-regression: Results for the meta-regression (Table 10) are limited to analyses that included three or more studies and was not possible according to breast cancer state [20, 23]. Adding Cheung (Singapore) decreased utility by 0.05 (p=0.14) and adding Ou (Taiwan) increased utility by 0.10 (p<0.001). Using the EQ5D five level version increased utility by 0.85 (p<0.001) and three level version decreased it by 0.07 (p=0.193). Using the Chinese three level value set or the Japanese value set decreased utility by 0.09 (p=0.007). Respondents with TNM stage III or IV had a reduction in utility by 0.002 (p<0.001). For every one percent increase in proportion of patients having chemotherapy, radiotherapy and surgery, utility increased by 0.003, 0.002 and 0.001 (p<0.001) respectively. Mean utility for university educated is 0.81 and by including Ou (Taiwan) this increased by 0.15 (p=0.54), excluding Taiwan it dropped by 0.44 (p<0.001). Utility according to income is 0.78 utility and if income is less than 30,000 Yuan per year then utility rises by 0.17 (p<0.001). For married women, mean utility is 0.67, adding Ou (Taiwan) increases this by 0.22 (p=0.43) and excluding it by 0.34 (p<0.001).

**Table 10:** Meta-regression of covariates for studies presenting summary data for all patients with breast cancer

Covariate	Level	Coefficient	95% CI	P value	Studies
Region	*China	0.82	0.79, 0.85		Li Wang Yang
	Singapore	-0.05	0.11, 0.02	0.141	Cheung
	Taiwan	0.10	0.04, 0.16	< 0.001	Ou
Version of EQ5D	EQ-5D-5L	0.85	0.80, 0.89	< 0.001	Li Yang Cheung Ou
	EQ-5D-3L	-0.07	-0.17, 0.03	0.193	Wang
Value set	Chinese 5L	0.87	0.84, 0.90	< 0.001	Li Yang Ou
	Chinese 3L	-0.09	-0.15, -0.02	0.007	Wang
	Japanese	-0.09	-0.16, -0.03	0.007	Cheung
TNM	Intercept	0.93	0.739		Li Wang Yang Ou
	Stage III or IV	-0.002	-0.008, 0.003	0.392	Li Wang Yang Ou
Chemotherapy	Intercept	0.70	0.68, 0.72		Li Wang Ou
	Prop of pts receiving Chemo	0.003	0.003, 0.004	< 0.001	Li Wang Ou
Radiotherapy	Intercept	0.80	0.76, 0.83		Li Wang Ou
	Prop of pts receiving Radio	0.002	0.001, 0.003	< 0.001	Li Wang Ou
Surgery	Intercept	0.79	0.75, 0.84		Li Wang Ou
	Prop of pts receiving Surgery	0.001	0.00, 0.002	< 0.001	Li Wang Ou
University	Intercept	0.81	0.66, 0.95		
	Including Taiwan	0.15	-0.33, 0.62	0.54	Li Yang Ou Cheung
	Excluding Taiwan	-0.44	-0.59, -0.28	< 0.001	Li Yang Cheung
Income	Intercept	0.78	0.76, 0.80		Li Yang Ou
	<30,000 Yuan / yr	0.17	0.09, 0.24	< 0.001	Li Yang Ou
Married	Intercept	0.67	0.21, 1.0		Li Yang Ou Cheung
	Including Taiwan	0.22	-0.32, 0.76	0.43	Li Yang Ou Cheung
	Excluding Taiwan	0.34	0.23, 0.46	< 0.001	Li Yang Cheung

*reference group

Extensive sensitivity analysis was undertaken and is shown in Table 11. The Li and Yang studies are most similar, both are conducted in China, use EQ5D5L and are valued with the China dataset, however the heterogeneity remains high at 85%. Heterogeneity increases to 97% when adding the Wang study (also conducted in China, but uses the 3-level version of the EQ5D) or the Ou study (different study region, but same version of EQ5D5L and same value set). Heterogeneity increases to 98% when adding the Cheung study conducted in Singapore, and 99% when all studies are combined.

**Table 11:** Sensitivity analysis, selective analysis for the five studies meeting the inclusion criteria

	Studies	Measure	Region	Value set	N	Mean	95% CI	Weight (%)	Estimate [95% CI]	I^2^(%)
1	Studies conducted in China, using EQ5D5L, valued by same China dataset (Luo 2017)									
	Li 2019	**EQ-5D-5L**	**China**	**China EQ5D5L, Luo 2017**	608	0.83	0.81, 0.84	51	0.84 [0.81, 0.87]	85
	Yang 2020	**EQ-5D-5L**	**China**	**China EQ5D5L, Luo 2017**	446	0.86	0.84, 0.87	48		
2	Studies using EQ5D5L in China									
	Li 2019	**EQ-5D-5L**	**China**	China EQ5D5L, Luo 2017	608	0.83	0.81, 0.84	33	0.82 [0.77, 0.87]	97
	Yang 2020	**EQ-5D-5L**	**China**	China EQ5D5L, Luo 2017	446	0.86	0.84, 0.87	32		
	Wang 2018	**EQ-5D-3L**	**China**	China EQ5D3L, Liu 2014	2626	0.78	0.77, 0.78	34		
3	Studies using EQ5D5L, valued by same China dataset (Luo 2017)									
	Li 2019	**EQ-5D-5L**	China	**China EQ5D5L, Luo 2017**	608	0.83	0.81, 0.84	33	0.87 [0.81, 0.92]	97
	Yang 2020	**EQ-5D-5L**	China	**China EQ5D5L, Luo 2017**	446	0.86	0.84, 0.87	33		
	Ou 2019	**EQ-5D-5L**	Taiwan	**China EQ5D5L, Luo 2017**	193	0.92	0.90, 0.93	33		
4	Studies using EQ5D5L									
	Li 2019	**EQ-5D-5L**	China	**China EQ5D5L, Luo 2017**	608	0.83	0.81, 0.84	25	0.84 [0.78, 0.90]	98
	Yang 2020	**EQ-5D-5L**	China	**China EQ5D5L, Luo 2017**	446	0.86	0.84, 0.87	24		
	Ou 2019	**EQ-5D-5L**	Taiwan	**China EQ5D5L, Luo 2017**	193	0.92	0.90, 0.93	25		
	Cheung 2014	**EQ-5D-5L**	Singapore	Japanese (Rabin)	238	0.77	0.75, 0.79	24		
5	All studies									
	Li 2019	EQ-5D-5L	China	China EQ5D5L, Luo 2017	608	0.83	0.81, 0.84	20	0.83 [0.77, 0.89]	99
	Yang 2020	EQ-5D-5L	China	China EQ5D5L, Luo 2017	446	0.86	0.84, 0.87	19		
	Ou 2019	EQ-5D-5L	Taiwan	China EQ5D5L, Luo 2017	193	0.92	0.90, 0.93	20		
	Cheung 2014	EQ-5D-5L	Singapore	Japanese (Rabin)	238	0.77	0.75, 0.79	19		
	Wang 2018	EQ-5D-3L	China	China EQ5D3L, Liu 2014	2626	0.78	0.77, 0.78	20		

## Discussion

We synthesised studies reporting health state utility values for Chinese women with breast cancer, for the purpose of parameterising a cost utility model. For all combinations of sensitivity analysis the range of utility values stayed between 0.82 when combining all five studies, to 0.87 when combining the two most closely related studies (Table 11). This 0.05 difference is less than the minimal clinically important difference reported by Pickard for all cancers (0.10 to 0.12 )[25]. As with all best practice in modelling, the uncertainty around the estimates produced in this meta-analysis should be explored in a probabilistic sensitivity analysis when incorporated into a cost utility model. As can be expected, the health state utility values for breast cancer patients overall (0.84) is lower than the general population in China (0.91 SD 0.18) [26]. Comparing the breast cancer stages, the random effects model utility for TNM 0, I and II was higher (0.85) than for TNM III (0.83) and TNM IV (0.73). However, the utility ranking did not match the severity of states (State P=0.84, State S=0.88, State R = 0.73, State M=0.78).

Our study has a number of strengths, firstly it is based on a systematic and transparent literature search strategy designed to have high specificity to identify utility elicited by EQ-5D. Previous work has highlighted the need for strict inclusion criteria to minimise heterogeneity and cautions against pooling utilities derived from different instruments [27, 28]. In keeping with this, we restricted our study to EQ5D alone, therefore results are highly valid for cost utility analyses requiring utility for women with different stages of breast cancer in China. The quality of the studies has been assessed and is considered suitable for pooling the data. Another strength is that four of the five studies use value sets derived from China population. Literature has highlighted between region difference in value sets and noted the importance of using the region specific value set [27]. This is especially apparent in the case of Cheung, which reports the lowest mean utility for women based on the Japanese value set. Comparing the value sets for China versus (vs.) Japan, the adjusted weighted average utility is higher for China (0.87 vs. 0.78 for 11112; 0.86 vs. 0.77 for 21111; 0.50 vs. 0.49 for 22222; 0.51 vs. 0.29 for 32211; -0.14 vs. -0.13 for 33333) [6, 29]. Therefore, including Cheung likely underestimates the utility of the general population in China. Ou is the outlier for region (Taiwan) and has the highest mean utility. Ou previously noted that the utility values were higher for Taiwan versus China. In addition, Ou includes only hormone receptor positive and human epidermal growth factor receptor 2 negative subtypes. When only the three China studies are included (Li, Yang, Wang) for random effects model the point estimate drops from 0.84 to 0.82 and the lower bound widens slightly from (0.81, 0.87) to (0.77,0.87).

The research should be interpreted along with it’s limitations. The first limitation is the high heterogeneity which is not uncommon with meta-analysis of EQ-5D and has been highlighted in the literature [16, 27, 30–32]. The interpretation of I^2^ is not well defined, as a guide, less than 40% may not be important; 30-60% indicates moderate heterogeneity, 50-90 indicates substantial heterogeneity and 75-100% considerable heterogeneity [33, 34]. Higgins has presented the case that “in relation to study effect sizes any amount of heterogeneity is acceptable, providing both that the predefined eligibility criteria for the metanalysis are sound and that the data are correct” [33]. Heterogeneity has been broadly described as clinical (variability in the participants, interventions, and outcomes) and methodological (study design, bias )[35]. In our study the methodological heterogeneity is well-defined and relates to the study region (China vs. Taiwan vs. Singapore), EQ-5D version (5L vs. 3L) and index value sets (EQ5D5L vs. EQ5D3L vs. Japanese). This explained heterogeneity is quantified in the meta-regression.

The clinical heterogeneity is more difficult to disentangle and contributes to the unexplained heterogeneity in our study. Synthesising the studies according to TNM status reduces the heterogeneity, although it remains considerable. Similarly, synthesising studies according to PSRM status further reduces heterogeneity notably to zero for State R. We believe this reflects the mix of treatments in the individual studies. For example the studies reported a range of treatments which are difficult to compare across studies because they reported different treatment combination, some of which are overlapping (not mutually exclusive) (see supplement). Chemotherapy for example has been identified as associated with poor quality of life (Ou). The study with the lowest proportion of patients having chemotherapy (alone) was Wang (up to 30%); Yang (up to 31%); Li (47%); Cheung (50%) and Ou (70%), (we note that these proportions of chemotherapy do not correspond to poorer quality of life values and further research is required to understand this relationship). Other studies have noted that chemotherapy is associated with poorer quality of life (Ou). Not only are different treatment combinations provided, but the treatment setting also varies, for example in the Li study, 100% were inpatients compared to 29% in Cheung and 16% in Yang. Inpatient/outpatient mix is not reported for Ou and Wang. We can hypothesise that inpatients are “sicker” than outpatients and hence have lower utility. Another source of variability that it is not possible to explore in our analysis is the breast cancer subtypes. Ou included 100% patients with breast cancer subtypes HR-positive/ HER2-negative; (see supplement); Li included 54% with ER/PR positive; Yang includes 80% HER positive patients. Chueng and Wang do not report the breast cancer subtypes. Literature suggests that patients have slightly different prognosis and outcomes than other breast cancer subtypes such as Luminal A and B. The subtypes are not provided for all studies and it was not possible to explore this variable for heterogeneity. The remaining heterogeneity likely reflects the diversity in duration since diagnosis (see supplement). The random effects model is likely to better account for the heterogeneity, however we wanted to present both sets of results so that the reader can see that there is not a large difference between the model estimates.

It is important to emphasise that in spite of the heterogeneity, the mean values do not vary greatly. A further limitation of our study is that the individual studies report mean and standard deviation, assuming normal distribution for their own analyses and the same assumption has been made for our analysis, however the ceiling effect of utility is well documented. The reader should be aware that utility measured with instruments other than EQ5D will likely give different results [36].

To the best of our knowledge there is no other meta-analysis of EQ-5D utility values specific to Chinese patients with breast cancer. Peasgood et al performed a meta-analysis of health-related quality of life in breast cancer patients more broadly, including all countries and all empirical health-related quality of life measuring instruments [37]. They identified 49 articles providing 476 utilities for breast cancer states including screening, prevention, adverse events, non-specific breast cancer, early and metastatic breast cancer. Utility values were pooled using ordinary least squares regression. The range of utilities from our study (0.82 to 0.87) were higher for early-stage breast cancer compared to Peasgood’s (0.66 to 0.78). The values for our study for TNM stages III and IV generally aligned with Peasgood’s findings (0.83 TNM III and 0.73 TNM IV versus Peasgood’s 0.72 and 0.80 for metastatic breast cancer). The Peasgood meta-analysis found that utility varied significantly according to valuation method [27]. There are no other other meta-analyses to our knowledge combining utility values for breast cancer.

## Conclusion

This study provides a meta-analysis of health state utility values measured by EQ5D by patients identifying as Chinese with breast cancer which may be used to inform cost utility models.

## Supplementary Information

**Additional file 1.**

## Data Availability

All data generated or analysed during this study are included in this published article and its supplementary information files.

## Abbreviations

AB: abstract
CI: confidence interval
COVID-19: corona virus disease 19
DARE: Database of abstracts of reviews of effectiveness
EQ5D: EuroQol 5-dimensions
EQ-5D-3L: EuroQol 5-dimensions three level
EQ-5D-5L: EuroQol 5-dimensions five level
HTA: Health technology assessment
NHS EED: National health service economic evaluation
NOS: Newcastle ottawa scale
PSRM: State P, first year after primary breast cancer; State R, first year after recurrence; State S, second and following years after primary breast cancer/recurrence; State M, metastatic breast cancer.
TI: title
TNM: Tumor Nodal Metastases
TTO: Time trade off
TW: Text word
PRISMA: Preferred Reporting Items for Systematic Reviews and Meta-Analyses
