# Supplementary material for: Meta-analysis of health state utility values measured by EuroQol 5-dimensions (EQ5D) questionnaire in Chinese women with breast cancer
Source: BMC Cancer. 2022 Jan 10;22:52. doi: 10.1186/s12885-021-09140-5 (PMC8744051; doi:10.1186/s12885-021-09140-5)
Supplement: Supplementary file 1 — Additional file 1. [file 12885_2021_9140_MOESM1_ESM.docx]

Meta-analysis of health state utility values measured by EuroQol 5-dimensions (EQ5D) questionnaire in Chinese women with breast cancer.

Rautenberg, Tamlyn^1^, Hodgkinson, Brent^1^, Zerwes, Ute^2^, Downes, Martin^1^.

1 Centre for Applied Health Economics, Griffith University

2 Assessment in Medicine GmbH, Germany

Supplement

Table of Contents

[1 Key features of included trials 3](#_Toc82544638)

[2 Sensitivity analysis 7](#_Toc82544639)

[3 Treatment 9](#_Toc82544640)

[4 Studies 10](#_Toc82544641)

[5 References 11](#_Toc82544642)

# Key features of included trials

| **Trial/Study** | **N** | **Design** | **Patient baseline characteristics and inclusion criteria** | **Key outcome(s)** |
| --- | --- | --- | --- | --- |
| Li 2019 | 608 | Single arm, recruitment from a single hospital,  China | \| **Characteristics** \| **N=608** \| \| --- \| --- \| \| Age in years: Mean ± SD \| 48.0 ± 9.6 \| \| Education n/N (%)  ≤ primary school  Secondary school  High school or technical  ≥ University \| 200/608 (32.9)  128/608 (21.1)  131/608 (21.5)  149/608 (24.5) \| \| Household Income in 2014 Yuan (x10,000) n/N (%)  <3  3 to 8  8-15  >15 \| 161/608 (26.5)  262/608 (43.1)  153/608 (25.2)  32/608 (5.3) \| \| Marital status n/N (%)  Married  Divorced/widowed  Single \| 539/608 (88.7)  42/608 (6.9)  27/608 (4.4) \| \| TNM stage n/N (%)  0-I  II  III  IV \| 175/608 (28.8)  142/608 (23.4)  218/608 (35.6)  73/608 (12.0) \| \| Disease duration in months n/N (%)  Mean ± SD  ≤12  13-36  37-60  ≥61 \| 38.2 ± 40.9  190/608 (31.3)  197/608 (32.4)  112/608 (18.4)  109/608 (17.9) \| \| Chemotherapy only n/N (%) \| 288/608 (47.4) \| \| ER/PR positive n/N (%) \| 329/608 (54.1) \|   Breast cancer patients who had undergone inpatient treatment | EQ-5D-5L  SF-6D, TTO |
| Ou 2019 | 193 | Cross sectional survey  Taiwan | \| **Characteristics** \| **N=193** \| \| --- \| --- \| \| Age in years: Mean ± SD \| 55.5 ± 9.0 \| \| Education n/N (%)  ≤ middle school  High school  ≥ University \| 36/193 (18.7)  76/192 (39.4)  81/193 (41.9) \| \| Individual income in Yuan (x10,000) n/N (%)  No income  <3  ≥3 \| 115/192 (59.9)  42/192 (21.9)  35/192 (18.2) \| \| Marital status n/N (%)  Married  Single/Divorced/widowed \| 154/192 (80.2)  38/192 (19.8) \| \| TNM stage n/N (%)  0  I  II  III  IV \| 22/193 (11.4)  51/193 (26.4)  71/193 (36.8)  38/193 (19.7)  4/193 (2.1) \| \| Disease duration in years n/N (%)  Mean ± SD  <3  3-6  7-9  >9 \| 5.4 ± 3.9  66/192 (34.4)  74/192 (38.5)  27/192 (14.1)  25/192 (13.0) \| \| Chemotherapy n/N (%) \| 146/193 (75.7) \|   Patients diagnosed with breast cancer with HR-positive/ HER2-negative subtype | EQ-5D-5L, EQ-VAS  QLQ-C30  QLQ-BR23 |
| Yang 2019 | 446 | Cross sectional survey  Tertiary oncology hospital,  China | \| **Characteristics** \| **N=446** \| \| --- \| --- \| \| Age in years: Mean ± SD \| 52.0 ± 8.8 \| \| Education n/N (%)  ≤ elementary  Junior high school  High school  ≥ University \| 144/446 (32.3)  150/446 (33.6)  88/446 (19.7)  64/446 (14.4) \| \| Household Income in Yuan (x10,000) n/N (%)  <3  3 to 8  8 to 15  ≥15 \| 234/446 (52.5)  148/446 (33.2)  45/446 (10.1)  19/446 (4.3) \| \| Marital status n/N (%)  Single  Married  Divorced  Widowed \| 7/446 (1.6)  411/446 (92.2)  14/446 (3.1)  14/446 (3.1) \| \| TNM stage n/N (%)  0  I  II  III  IV \| 17/446 (3.8)  72/446 (16.4)  224/446 (50.2)  99/446 (22.2)  34/446 (7.6) \| \| Disease duration in months n/N (%)  ≤12  13-36  37-60  ≥ 61 \| 133/446 (29.8)  147/446 (33.0)  78/446 (17.5)  88/446 (19.7) \| \| Chemotherapy n/N (%) \| 409/446 (91.7) \| \| HER positive n/N (%) \| 355/446 (79.6) \|   Breast cancer patients diagnosed by pathology or clinical tests, >18 years, no mental disorders | EQ-5D-5L  FACT-B  EQ-VAS |
| Wang 2018 | 2626 | Cross sectional survey  12 Provinces in China  Poster only | \| **Characteristics** \| **N=446** \| \| --- \| --- \| \| Age in years: Mean \| 49.1 \|   Patients with breast cancer | EQ-5D  EQ-VAS |
| Cheung 2014 | 238 | Cross sectional survey  2 cancer centres,  Singapore | \| **Characteristics** \| **N=238** \| \| --- \| --- \| \| Age in years: Mean ± SD \| 51.3 ± 9.7 \| \| Education n/N (%)  ≤ primary  Secondary  Postsecondary \| 48/238 (20.3)  112/238 (47.3)  77/238 (32.5) \| \| Race  Chinese  Malay  Indian  Other \| 193/238 (81.1)  23/238 (9.7)  18/238 (7.6)  4/238 (1.7) \| \| Marital status n/N (%)  Single  Married  Divorced  Widowed \| 47/238 (19.8)  168/238 (70.9)  12/238 (5.1)  10/238 (4.2) \| \| Patient assessed performance status^a^ n/N (%)  0  1  2  3 or 4 \| 97/238 (40.8)  115/238 (48.3)  17/238 (7.1)  9/238 (3.8) \| \| Evidence of disease n/N (%) \| 118/236 (50.0) \| \| Chemotherapy n/N (%) \| 100/238 (42.0) \|   Breast cancer patients diagnosed by histopathology, ≥ 21 years, no mental disorders | EQ-5D-5L  FACT-B |

^a^ describes the cancer patient from five options ranging from 0 (without symptoms) to 4 (bedridden). Note that separate results reported for precancer and cancer patients – only cancer patients results have been used for this analysis.

# Sensitivity analysis

| **Comp** | **Studies** | **Measure** | **Country** | **Value set** | **N** | **Mean** | | **95% CI** | **Weight (%)** | **Estimate [95% CI]** | **I squared (%)** |
| --- | --- | --- | --- | --- | --- | --- | --- | --- | --- | --- | --- |
| 1 | Studies conducted in China, using EQ5D5L, valued by same China dataset (Luo 2017) | | | | | | | | | | |
|  | Li 2019 | **EQ-5D-5L** | **China** | **China EQ5D5L, Luo 2017** | 608 | 0.830 | | 0.816, 0.844 | 51.5 | 0.845 [0.815, 0.874] | 85.1 |
|  | Yang 2020 | **EQ-5D-5L** | **China** | **China EQ5D5L, Luo 2017** | 446 | 0.860 | | 0.842, 0.878 | 48.5 |  |  |
|  | | | | | | | | | | | |
| 2 | Studies using EQ5D5L, valued by same China dataset (Luo 2017) | | | | | | | | | | |
|  | Li 2019 | **EQ-5D-5L** | China | **China EQ5D5L, Luo 2017** | 608 | | 0.830 | 0.816, 0.844 | 33.4 | 0.870 [0.813, 0.927] | 97.8 |
|  | Yang 2020 | **EQ-5D-5L** | China | **China EQ5D5L, Luo 2017** | 446 | | 0.860 | 0.842, 0.878 | 33.6 |  |  |
|  | Ou 2019 | **EQ-5D-5L** | Taiwan | **China EQ5D5L, Luo 2017** | 193 | | 0.920 | 0.907, 0.933 | 33.0 |  |  |
|  | | | | | | | | | | | |
| 3 | Studies using EQ5D5L | | | | | | | | | | |
|  | Li 2019 | **EQ-5D-5L** | China | **China EQ5D5L, Luo 2017** | 608 | | 0.830 | 0.816, 0.844 | 25.1 | 0.847 [0.788, 0.906] | 98.2 |
|  | Yang 2020 | **EQ-5D-5L** | China | **China EQ5D5L, Luo 2017** | 446 | | 0.860 | 0.842, 0.878 | 24.9 |  |  |
|  | Ou 2019 | **EQ-5D-5L** | Taiwan | **China EQ5D5L, Luo 2017** | 193 | | 0.920 | 0.907, 0.933 | 25.2 |  |  |
|  | Cheung 2014 | **EQ-5D-5L** | Singapore | Japanese (Rabin) | 238 | | 0.777 | 0.756, 0.798 | 24.7 |  |  |
|  | | | | | | | | | | | |
| 4 | Studies using EQ5D5L in China | | | | | | | | | | |
|  | Li 2019 | **EQ-5D-5L** | **China** | China EQ5D5L, Luo 2017 | 608 | | 0.830 | 0.816, 0.844 | 33.2 | 0.823[0.775, 0.872] | 97.8 |
|  | Yang 2020 | **EQ-5D-5L** | **China** | China EQ5D5L, Luo 2017 | 446 | | 0.860 | 0.842, 0.878 | 32.8 |  |  |
|  | Wang 2018 | **EQ-5D-3L** | **China** | China EQ5D3L, Liu 2014 | 2626 | | 0.781 | 0.774, 0.788 | 34.0 |  |  |
|  | | | | | | | | | | | |
| 5 | All studies | | | | | | | | | | |
|  | Li 2019 | EQ-5D-5L | China | China EQ5D5L, Luo 2017 | 608 | | 0.830 | 0.816, 0.844 | 20.0 | 0.834 [0.776, 0.891] | 99.0 |
|  | Yang 2020 | EQ-5D-5L | China | China EQ5D5L, Luo 2017 | 446 | | 0.860 | 0.842, 0.878 | 19.9 |  |  |
|  | Ou 2019 | EQ-5D-5L | Taiwan | China EQ5D5L, Luo 2017 | 193 | | 0.920 | 0.907, 0.933 | 20.1 |  |  |
|  | Cheung 2014 | EQ-5D-5L | Singapore | Japanese (Rabin) | 238 | | 0.777 | 0.756, 0.798 | 19.8 |  |  |
|  | Wang 2018 | EQ-5D-3L | China | China EQ5D3L, Liu 2014 | 2626 | | 0.781 | 0.774, 0.788 | 20.2 |  |  |
|  | | | | | | | | | | | |
| 6 | Studies reporting Utility at less than 36 months after diagnosis | | | | | | | | | | |
|  | Li 2019 | **EQ-5D-5L** | China | **China EQ5D5L, Luo 2017** | 197 | | 0.830 | 0.816, 0.844 | 49.5 | 0.880 [0.802, 0.959 | 95.9 |
|  | Ou 2019 | **EQ-5D-5L** | Taiwan | **China EQ5D5L, Luo 2017** | 66 | | 0.920 | 0.907, 0.933 | 50.5 |  |  |
|  | | | | | | | | | | | |
| 7 | Studies reporting Utility at less than 60 to 72 months after diagnosis | | | | | | | | | | |
|  | Li 2019 | **EQ-5D-5L** | China | **China EQ5D5L, Luo 2017** | 112 | | 0.850 | 0.813, 0.887 | 47.6 | 0.887 [0.818, 0.955] | 90.2 |
|  | Ou 2019 | **EQ-5D-5L** | Taiwan | **China EQ5D5L, Luo 2017** | 74 | | 0.920 | 0.898, 0.942 | 52.4 |  |  |
|  | | | | | | | | | | | |
| 8 | Studies reporting Utility at greater than 60 months after diagnosis | | | | | | | | | | |
|  | Li 2019 | **EQ-5D-5L** | China | **China EQ5D5L, Luo 2017** | 109 | | 0.810 | 0.778, 0.842 | 50.3 | 0.875 [0.747, 1.00] | 96.2 |
|  | Ou 2019 | **EQ-5D-5L** | Taiwan | **China EQ5D5L, Luo 2017** | 27 | | 0.940 | 0.902, 0.978 | 49.7 |  |  |
|  | | | | | | | | | | | |
| 9 | Chemotherapy | | | | | | | | | | |
|  | Li 2019 | **EQ-5D-5L** | China | **China EQ5D5L, Luo 2017** | 288 | | 0.860 | 0.840, 0.880 | 33.2 | 0.856 [0.790, 0.922] | 97.6 |
|  | Ou 2019 | **EQ-5D-5L** | Taiwan | **China EQ5D5L, Luo 2017** | 135 | | 0.910 | 0.893, 0.927 | 33.4 |  |  |
|  | Wang 2018 | EQ-5D-3L | China | China EQ5D3L, Liu 2014 | 689 | | 0.799 | 0.782, 0.816 | 33.4 |  |  |
|  | | | | | | | | | | | |
| 10 | Radiotherapy | | | | | | | | | | |
|  | Li 2019 | **EQ-5D-5L** | China | **China EQ5D5L, Luo 2017** | 14 | | 0.790 | 0.732, 0.848 | 32.3 | 0.825 [0.693, 0.957 | 97.8 |
|  | Ou 2019 | **EQ-5D-5L** | Taiwan | **China EQ5D5L, Luo 2017** | 125 | | 0.930 | 0.914, 0.946 | 34.2 |  |  |
|  | Wang 2018 | EQ-5D-3L | China | China EQ5D3L, Liu 2014 | 193 | | 0.75 | 0.72, 0.76 | 33.5 |  |  |
|  | | | | | | | | | | | |
| 11 | Surgery | | | | | | | | | | |
|  | Li 2019 | **EQ-5D-5L** | China | **China EQ5D5L, Luo 2017** | 221 | | 0.73 | 0.704, 0.756 | 33.3 | 0.797 [0.686, 0.909] | 98.4 |
|  | Ou 2019 | **EQ-5D-5L** | Taiwan | **China EQ5D5L, Luo 2017** | 179 | | 0.925 | 0.896, 0.954 | 33.1 |  |  |
|  | Wang 2018 | EQ-5D-3L | China | China EQ5D3L, Liu 2014 | 491 | | 0.738 | 0.720, 0.756 | 33.6 |  |  |
|  | | | | | | | | | | | |
| 12 | Concurrent chemoradiotherapy | | | | | | | | | | |
|  | Li 2019 | **EQ-5D-5L** | China | **China EQ5D5L, Luo 2017** | 26 | | 0.65 | 0.596, 0.704 | 48.4 | 0.707 [0.599, 0.814] | 90.7 |
|  | Wang 2018 | EQ-5D-3L | China | China EQ5D3L, Liu 2014 | 47 | | 0.76 | 0.714, 0.790 | 51.6 |  |  |

# Treatment

| Study/sample size | Treatment | Count | Percentage |
| --- | --- | --- | --- |
| Li n=608 | Chemotherapy only | 288 | 47% |
|  | Endocrine therapy | 73 | 12% |
|  | Surgery & Chemotherapy | 69 | 11% |
|  | Re‐examination | 46 | 8% |
|  | Radio‐& Chemotherapy | 26 | 4% |
|  | Targeted therapy | 22 | 4% |
|  | Surgery | 21 | 3% |
|  | Radiotherapy | 14 | 2% |
|  | Others | 49 | 8% |
|  | **Total** |  | **100%** |
| Yang n=466 | Endocrine therapy | 307 | 69% |
|  | No endocrine therapy | 139 | 31% |
|  | **Total** |  | **100%** |
| Ou n=193 | Tamoxifen | 107 | 55% |
|  | Aromatase inhibitor | 79 | 41% |
|  | Chemotherapy | 135 | 70% |
|  | Radiotherapy | 125 | 65% |
|  | Mastectomy partial | 71 | 37% |
|  | Mastectomy total | 108 | 56% |
|  | **Total** |  | **324%** |
| Wang n=2626 | surgery | 491 | 20% |
|  | symptomatic treatment | 106 | 0% |
|  | radiotherapy | 193 | 10% |
|  | chemotherapy | 689 | 30% |
|  | surgery post operative | 849 | 30% |
|  | neoadjuvant, chemotherapy and surgery | 179 | 10% |
|  | chemo and radio | 47 | 0% |
|  | other | 19 | 0% |
|  | **Total** |  | **100%** |
| Cheung n=238 | treatment adjuvant, curative, hormone | 112 | 50% |
|  | palliative | 78 | 30% |
|  | no treatment | 45 | 20% |
|  | **Total** |  | **100%** |

# Studies

The following two manuscripts report the same study data.

Yang, Q., et al., Mapping function from FACT-B to EQ-5D-5 L using multiple modelling approaches: data from breast cancer patients in China. Health Qual Life Outcomes, 2019. 17(1): p. 153.

Yang, Q., X. Yu, and W. Zhang, Health variations among breast-cancer patients from different disease states: evidence from China. BMC Health Serv Res, 2020. 20(1): p. 1033.

The following two manuscripts report the same study data.

Cheung, Y.B., et al., Mapping the functional assessment of cancer therapy-breast (FACT-B) to the 5-level EuroQoL Group's 5-dimension questionnaire (EQ-5D-5L) utility index in a multi-ethnic Asian population. Health Qual Life Outcomes, 2014. 12: p. 180.

Lee, C., F.; Luo, N, N et al. Comparison of the measurement properties between a short and generic instrument, the 5-level EuroQoL Group's 5-dimension (EQ-5D-5L) questionnaire, and a longer and disease-specific instrument, the Functional Assessment of Cancer Therapy—Breast (FACT-B), in Asian breast cancer patients Quality of life research, 09/2013, Volume 22, Issue 7

# References

Sung, H., et al., Global Cancer Statistics 2020: GLOBOCAN Estimates of Incidence and Mortality Worldwide for 36 Cancers in 185 Countries. CA Cancer J Clin, 2021. 71(3): p. 209-249.

Cao, W., et al., Changing profiles of cancer burden worldwide and in China: a secondary analysis of the global cancer statistics 2020. Chin Med J (Engl), 2021. 134(7): p. 783-791.

Chen, W., et al., Cancer statistics in China, 2015. CA Cancer J Clin, 2016. 66(2): p. 115-32.

Liu, G., China Guidelines for Pharmacoeconomic Evaluations 2020 Edition, ed. G. Liu. 2020, Beijing China.

China Guidelines for Pharmacoeconomic Evaluations 2011; Available from: ISPOR website.

Luo, N., et al., Estimating an EQ-5D-5L Value Set for China. Value Health, 2017. 20(4): p. 662-669.

Lee, C.F., et al., The English and Chinese versions of the five-level EuroQoL Group's five-dimension questionnaire (EQ-5D) were valid and reliable and provided comparable scores in Asian breast cancer patients. Support Care Cancer, 2013. 21(1): p. 201-9.

Kim, S.-H., et al., Validity and reliability of EQ-5D-3L for breast cancer patients in Korea. Health and Quality of Life Outcomes, 2015. 13(1): p. 203.

Kimman, M.L., et al., Responsiveness of the EQ-5D in breast cancer patients in their first year after treatment. Health and quality of life outcomes, 2009. 7: p. 11-11.

Saramago, P., A. Manca, and A.J. Sutton, Deriving input parameters for cost-effectiveness modeling: taxonomy of data types and approaches to their statistical synthesis. Value Health, 2012. 15(5): p. 639-49.

Wells, G.S., B. O'Connell, D. Peterson, J. Welch, V. Losos, M. Tugwell, P. The Newcastle-Ottawa Scale (NOS) for assessing the quality of nonrandomised studies in meta-analyses. 2020 [cited 2020 26.12.2020]; Available from: http://www.ohri.ca/programs/clinical_epidemiology/oxford.asp.

Balk, E.M., et al., AHRQ Comparative Effectiveness Reviews, in Venous Thromboembolism Prophylaxis in Major Orthopedic Surgery: Systematic Review Update. 2017, Agency for Healthcare Research and Quality (US): Rockville (MD).

Cortegiani, A., et al., Update I. A systematic review on the efficacy and safety of chloroquine/hydroxychloroquine for COVID-19. J Crit Care, 2020. 59: p. 176-190.

Moher, D., et al., Preferred reporting items for systematic reviews and meta-analyses: the PRISMA statement. Bmj, 2009. 339: p. b2535.

Moher, D., et al., Preferred reporting items for systematic review and meta-analysis protocols (PRISMA-P) 2015 statement. Syst Rev, 2015. 4(1): p. 1.

Petrou, S., J. Kwon, and J. Madan, A Practical Guide to Conducting a Systematic Review and Meta-analysis of Health State Utility Values. Pharmacoeconomics, 2018. 36(9): p. 1043-1061.

Schwarzer, G. Meta: an R package for meta-analysis. 2007 [cited 2020 27.12.2020]; Available from: https://cran.rstudio.org/doc/Rnews/Rnews_2007-3.pdf#page=40.

Higgins, J.P., et al., Measuring inconsistency in meta-analyses. Bmj, 2003. 327(7414): p. 557-60.

Yang, Q., et al., Mapping function from FACT-B to EQ-5D-5 L using multiple modelling approaches: data from breast cancer patients in China. Health Qual Life Outcomes, 2019. 17(1): p. 153.

Yang, Q., X. Yu, and W. Zhang, Health variations among breast-cancer patients from different disease states: evidence from China. BMC Health Serv Res, 2020. 20(1): p. 1033.

Cheung, Y.B., et al., Mapping the functional assessment of cancer therapy-breast (FACT-B) to the 5-level EuroQoL Group's 5-dimension questionnaire (EQ-5D-5L) utility index in a multi-ethnic Asian population. Health Qual Life Outcomes, 2014. 12: p. 180.

Wang, L., et al., Health-related quality of life and utility scores of patients with breast neoplasms in China: A multicenter cross-sectional survey. Breast, 2018. 39: p. 53-62.

Li, S., et al., Which approach is better in eliciting health state utilities from breast cancer patients? Evidence from mainland China. Eur J Cancer Care (Engl), 2019. 28(2): p. e12965.

Ou, H.T., et al., Health-related quality of life associated with different cancer treatments in Chinese breast cancer survivors in Taiwan. Eur J Cancer Care (Engl), 2019. 28(4): p. e13069.

Pickard, A.S., M.P. Neary, and D. Cella, Estimation of minimally important differences in EQ-5D utility and VAS scores in cancer. Health Qual Life Outcomes, 2007. 5: p. 70.

Wang, H.M., et al., Validation of the EQ-5D in a general population sample in urban China. Qual Life Res, 2012. 21(1): p. 155-60.

Peasgood, T. and J. Brazier, Is Meta-Analysis for Utility Values Appropriate Given the Potential Impact Different Elicitation Methods Have on Values? Pharmacoeconomics, 2015. 33(11): p. 1101-5.

Sturza, J., A review and meta-analysis of utility values for lung cancer. Med Decis Making, 2010. 30(6): p. 685-93.

Tsuchiya, A., et al., Estimating an EQ-5D population value set: the case of Japan. Health Econ, 2002. 11(4): p. 341-53.

Peasgood, T., et al., An updated systematic review of Health State Utility Values for osteoporosis related conditions. Osteoporos Int, 2009. 20(6): p. 853-68.

Doth, A.H., et al., The burden of neuropathic pain: a systematic review and meta-analysis of health utilities. Pain, 2010. 149(2): p. 338-44.

McLernon, D.J., J. Dillon, and P.T. Donnan, Health-state utilities in liver disease: a systematic review. Med Decis Making, 2008. 28(4): p. 582-92.

Higgins, J.P., Commentary: Heterogeneity in meta-analysis should be expected and appropriately quantified. Int J Epidemiol, 2008. 37(5): p. 1158-60.

Patsopoulos, N.A., E. Evangelou, and J.P. Ioannidis, Sensitivity of between-study heterogeneity in meta-analysis: proposed metrics and empirical evaluation. Int J Epidemiol, 2008. 37(5): p. 1148-57.

Haidich, A.B., Meta-analysis in medical research. Hippokratia, 2010. 14(Suppl 1): p. 29-37.

Rautalin, M., et al., Health-related quality of life in different states of breast cancer - comparing different instruments. Acta Oncol, 2018. 57(5): p. 622-628.

Peasgood, T.W., S. Brazier, J. HEDS Discussion Paper 10/15. A review and meta-analysis of health state utility values in breast cancer. 2010 27.12.2020]; Available from: https://mpra.ub.uni-muenchen.de/29950/.
